# Preventing Pilonidal Sinus Recurrence With Laser Hair Epilation: A Systematic Review and Meta-Analysis of Randomized Controlled Trials

**DOI:** 10.7759/cureus.62807

**Published:** 2024-06-21

**Authors:** Neil Muscat, Apurv Gupta, Mohammed Arifuzaman, Firuza Soxibova

**Affiliations:** 1 General Surgery, Bolton NHS Foundation Trust, Bolton, GBR; 2 General Surgery, North Manchester General Hospital, Manchester, GBR; 3 General Surgery, Royal Albert Edward Infirmary, Wrightington, Wigan and Leigh (WLL) NHS Foundation Trust, Wigan, GBR

**Keywords:** disease, removal, epilation, hair, laser, recurrence, sinus, pilonidal

## Abstract

Pilonidal sinus disease (PSD) is a common condition associated with significant morbidity and healthcare costs. High recurrence rates still pose a considerable challenge in managing PSD, with no universally accepted guideline in place to guide management. Laser hair epilation offers a way to reduce recurrence rates with reports within the current literature demonstrating positive outcomes compared to alternative approaches. This review was conducted by the Preferred Reporting Items for Systematic Reviews and Meta-Analysis (PRISMA) statement standards. The primary outcome measure was the recurrence rate of PSD at a minimum of one year following laser hair epilation. The electronic databases of MEDLINE, EMBASE, CINAHL, Google Scholar, PubMed, and Cochrane Central Register of Controlled Trials (CENTRAL) were searched. OpenMeta Analyst (Brown University School of Public Health, Providence, RI) software was used for data synthesis. Three randomized controlled trials met the inclusion criteria with laser hair epilation treatments offering a significant reduction in PSD recurrence rates on odds ratio analysis: 0.319 ( 0.160, 0.636), *P*-value = 0.0001. Secondary outcomes involving patient disability days, caregiver disability days, health-related quality-of-life (HRQOL) scores, healthcare satisfaction scores, and perceived stigma were discussed qualitatively. The authors offer a decisive recommendation in favor of laser hair epilation in PSD; however, they recommend further high-quality trials to investigate the ideal timing and frequency of laser hair epilation sessions.

## Introduction and background

Pilonidal sinus disease (PSD) is a persistent inflammatory condition first described in 1833 by O.H. Mayo distinguished by the existence of a cyst, abscess, or sinus tract in the intergluteal cleft, adjacent to the coccyx. This condition is associated with significant morbidity and healthcare costs and predominantly impacts individuals in their youth, showing a greater occurrence among males with an estimated incidence of 26 per 100,000 [1].

Due to the high recurrence rates associated with PSD, the condition was initially thought to be congenital; however, it is now generally accepted to be acquired in etiology [2]. Karydakis proposed that three core elements are involved in the pathogenesis of PSD: hair acting as a foreign body, the force needed to cause its implantation, and skin vulnerability essential to allow the hair to reach the level of the natal cleft. This theory is substantiated by the frequent discovery of hair in sinus tracts and cysts detected during surgical interventions. Other contributing elements encompass deep natal clefts, obesity, excessive body hair, and substandard hygiene practices. Friction and pressure resulting from prolonged sitting or tight apparel exacerbate the situation by facilitating hair penetration and entrapping debris in the natal cleft [3].

The long-term (> 5 years) recurrence rate for PSD is estimated to be 22% with 71% of recurrences occurring within 4 years of the primary surgery and an associated median recurrence-free interval of 1.8 years [4]. Early recurrence is caused by the inability to identify one or more sinuses during surgery, while late recurrence is usually due to secondary infection, hair or debris not removed during surgery, inadequate wound care, or lack of attention to depilation [5]. 

A variety of surgical options are currently employed. The conventional approach of excision and primary closure entails the removal of the sinus tract, followed by primary closure of the wound using sutures. Although this method promotes faster healing, it is associated with relatively high rates of recurrence. Surgeons strive to eliminate all affected tissue and flatten the natal cleft to minimize hair accumulation and friction. Alternatively, excision and healing with secondary intention entail the removal of the sinus followed by allowing the wound to heal on its own without closure. Despite the longer healing time and the need for more extensive wound care, this technique exhibits lower recurrence rates in comparison to primary closure. This is attributed to its ability to minimize the likelihood of any remaining disease after the procedure [3,5].

Flap techniques, such as the rhomboid (Limberg) flap, Karydakis flap, and Bascom cleft lift, are employed to reshape the area to reduce the depth of the natal cleft and redistribute tension away from the midline. These procedures not only reduce hair re-entry and friction but also enhance blood supply to the area, leading to improved healing and significantly reduced recurrence rates. Recent advancements have introduced minimally invasive techniques such as endoscopic pilonidal sinus treatment (EPSiT) and video-assisted ablation of pilonidal sinus (VAAPS). These modern methods use endoscopic instruments to remove the sinus tract with minimal disruption to surrounding tissues, resulting in faster recovery times and a lower risk of recurrence [5].

The provision of postoperative care is essential in mitigating the risk of recurrence following a surgical procedure. Patients are typically counseled to take meticulous care of their wounds, maintain cleanliness, and avoid prolonged periods of sitting. Moreover, patients may be recommended to shave the area or undergo laser treatments to prevent hair from obstructing their sinus passages. Educating patients on the importance of these measures is critical for facilitating a smooth recovery process and reducing the chances of recurrence [5]. 

Continuous research efforts are directed toward improving the understanding of the pathophysiology of PSD and developing more efficient treatment strategies. Genetic studies are actively investigating potential hereditary factors that may increase the susceptibility of individuals to PSD. Additionally, there is a strong emphasis on the development of non-surgical treatments to better manage PSD with fewer complications. The use of biologics and novel agents to regulate the inflammatory response in PSD represents a promising area of research. These treatments have the potential to lessen the need for surgical intervention and improve long-term outcomes. Additionally, investigations into wound care technology advancements, like negative pressure wound therapy (NPWT), are ongoing to determine their effectiveness in enhancing healing and reducing recurrence.

The reduction of hair in the natal cleft is thought to reduce recurrence by disrupting one of the 3 factors described by Karydakis in 1992. Techniques such as cream depilation and traditional shaving with a standard razor are associated with a low compliance rate as the natal cleft is hard to access and shaving produces side effects such as rashes and cuts [6]. Lasers offer a safe and effective method of hair removal by targeting melanin in the hair shaft, follicular epithelium, and hair matrix by emitting light with wavelengths between 600 and 1200 nm, which is selectively absorbed by melanin while sparing surrounding tissue. Laser hair removal also offers a more permanent reduction in hair growth when compared to traditional methods which reduces the risk of hair penetrating the skin and forming new sinuses. Additionally, lasers are more effective in areas of dense hair growth as they can specifically target course, dark hairs [6]. The number of sessions needed may differ depending on various factors, including the thickness of hair, the type of skin, and the specific laser technology employed. To achieve optimal results and reduce any potential side effects, it is essential to recommend the avoidance of sun exposure and adherence to skincare regimes. Adverse effects are minimal and include mild discomfort in addition to temporary changes in skin pigmentation in patients with darker Fitzpatrick scale skin types [7].

A variety of clinical studies have been conducted to define the effect of laser hair removal on pilonidal sinus recurrence however data from randomized controlled trials (RCT) has been sparse. The authors aim to determine the effectiveness of laser hair removal in preventing PSD recurrence by conducting a systemic review and meta-analysis, accounting for recent results achieved under RCT conditions.

## Review

Methodology

This systematic review and meta-analysis was conducted by the Preferred Reporting Items for Systematic Reviews and Meta-Analysis (PRISMA) statement standard [8].

Eligibility Criteria

All RCTs investigating the effect of laser hair removal on PSD were included. Laser hair epilation was the intervention of interest and routine wound care or conventional hair removal techniques were the comparators. All patients were included, irrespective of age, gender, or comorbidity status. Articles not reported in English were excluded. Only full-text articles were included.

Primary and Secondary Outcome Measures

The primary outcome measure was the recurrence rate of PSD at a minimum of one year following laser hair epilation. Secondary outcomes such as wound site infection symptoms, wound separation, patient disability days, caregiver disability days, HRQOL scores, healthcare satisfaction scores, and perceived stigma were discussed qualitatively.

Literature Search Strategy

Three authors NDM, AG, and FS independently searched the electronic databases of MEDLINE, EMBASE, CINAHL, Google Scholar, PubMed, and Cochrane Central Register of Controlled Trials (CENTRAL). The last search was conducted on April 10, 2024. The search strategy utilized the Thesaurus headings, search operators, and limits of each of the aforementioned databases. Search terms for our intervention of interest involved “pilonidal sinus,” “laser,” “hair,” “removal,” and “epilation." All terms were merged utilizing adjuncts of “or” and “and." To increase our search, the bibliographic lists of the relevant articles were screened.

Selection of Studies

Article titles and abstracts were retrieved from the literature search by two authors, NDM and AG. The full texts of articles that met the eligibility criteria were retrieved and reviewed.

Data Extraction and Management 

To review interventions, a data extraction table was created using Microsoft Excel in line with Cochrane's data collection form. Subsequently, the table was adjusted after being pilot-tested in randomly selected articles. It included study-related data: study name, year of publication, study design, number of patients in the intervention and control group, one-year recurrence rates of laser and control groups, in addition to secondary outcome measures. NDM and AG, two authors in the series collected the results and recorded them; disputes were resolved through discussion with an independent author FS.

Data Synthesis

Data were synthesized using OpenMeta Analyst (Brown University School of Public Health, Providence, RI) software. Using two independent authors, NDM and AG, the extracted data were entered into the software. The analysis involved utilizing a binary fixed-effects model with a generic inverse variance function being applied to account for cases of moderate to high heterogeneity and utilizing an odds ratio (OR) on the dichotomous data. The result about the primary outcome was reported on a forest plot with 95% confidence intervals (CIs).

Assessment of Heterogeneity

The data were assessed for heterogeneity using the Cochrane Q test (X2) and through the calculation of the I2 value. The studies yielded a statistically significant heterogeneity (I^2 = 77.12%, *P *= 0.013), which was successfully accounted for by using an inverse variance function (I^2 = 0%, *P *= 0.013).

Methodological Quality and Risk-of-Bias Assessment

For publications that satisfied the qualifying requirements, two authors, NDM and AG, independently evaluated the methodological quality and the risk of bias using the Cochrane Collaboration's tool, which classified RCTs as having a low, high, or unclear risk of bias (Table 1).

**Table 1:** Cochrane Collaboration’s tool. Assessment of risk of bias for the randomized controlled trials utilizing the Cochrane Collaboration’s tool.

Studies	Bias	Authors' judgment	Support for judgment	
Minneci et al. [9]	Selection bias	-	-	
	Random sequence generation	Low	Block randomization	
	Allocation concealment	High	Not hidden from patients	
	Reporting bias	-	-	
	Selective reporting	Low	All outcomes discussed	
	Other bias	-	-	
	Other sources of bias	Low	No other bias detected	
	Performance bias	-	-	
	Blinding	High	Assessor blinding unclear. Patients not blinded	
	Detection bias	-	-	
	Blinding (outcome assessment)	Unclear	Does not state if assessors were blinded	
	Attrition bias	-	-	
	Incomplete outcome data	Low	All patient attritions and exclusions accounted for	
Demircan et al. [10]	Selection bias	-	-	
	Random sequence generation	Low	Simple randomization	
	Allocation concealment	High	Not hidden from patients	
	Reporting bias	-	-	
	Selective reporting	Low	All outcomes presented	
	Other bias	-	-	
	Other sources of bias	Low	No other bias detected	
	Performance bias	-	-	
	Blinding [participants and personnel]	High	Participants and most personnel not blinded	
	Detection bias	-	-	
	Blinding [outcome assessment]	Low	One assessor blinded	
Ghnnam and Hafez [11]	Selection bias	-	-	
	Random sequence generation	Low	Simple randomization	
	Allocation concealment	High	Not hidden from patients	
	Reporting bias	-	-	
	Selective reporting	Low	All outcomes discussed	
	Other bias	-	-	
	Other sources of bias	Low	No other bias detected	
	Performance bias	-	-	
	Blinding [participants and personnel]	High	Participants and personnel not blinded	
	Detection bias	-	-	
	Blinding [outcome assessment]	Unclear	Does not state if assessors were blinded	
	Attrition bias	-	-	
	Incomplete outcome data	Low	All patients included in results	

Results

**Figure 1:**
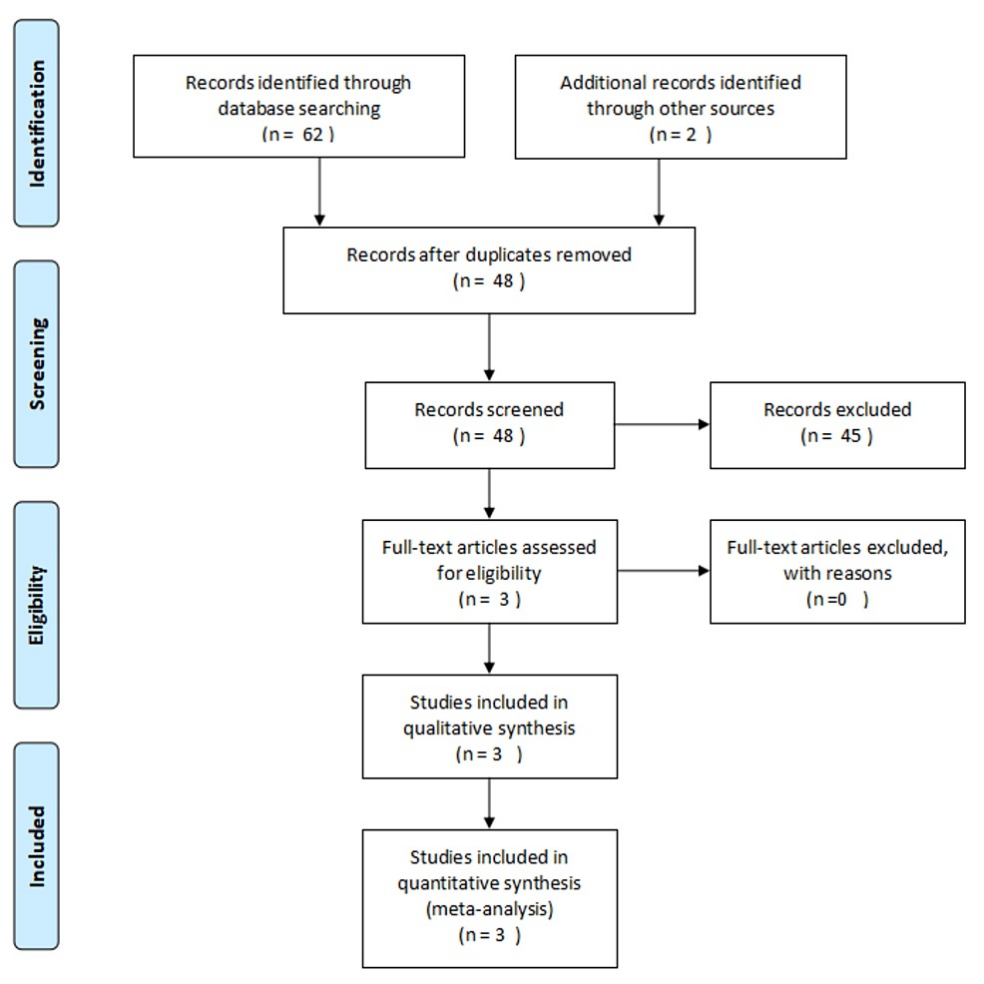
PRISMA flow diagram detailing the search and selection processes applied during the literature review PRISMA, Preferred Reporting Items for Systematic Reviews and Meta-Analysis

After adhering to PRISMA recommendations, three papers were identified that were eligible for inclusion in our meta-analysis (Figure 1).

*Primary Outcome: *PSD* Recurrence*

**Figure 2:**
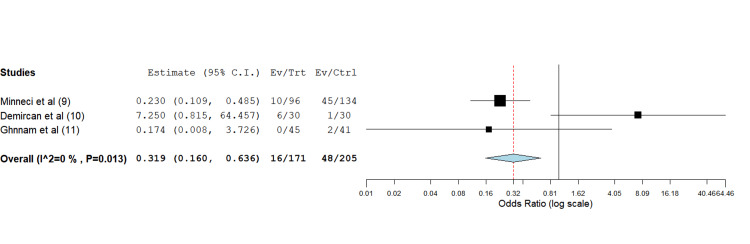
Odds ratio estimate 0.319 (lower bound 0.160, upper bound 0.636), P-value = 0.001.

In total, three RCTs between 2011 and 2023 reported PSD recurrence. A significant difference (*P* = 0.0001) was identified on an OR analysis: 0.319 ( 0.160, 0.636), utilizing a binary fixed-effect model, with a lower incidence of recurrence reported following laser hair epilation (Figure 2). An inverse variance function was implemented to account for heterogeneity.

The prospective RCT conducted by Minecci et al. [9] involved a total of 302 participants, comprising adolescents and young adults aged between 12 and 21 years with a diagnosis of PSD. Patients with an actively inflamed pilonidal sinus and a history of photosensitivity were excluded from the study. The researchers contrasted the efficacy of mechanical or chemical hair removal as a standalone method in one group, with the incorporation of laser hair removal therapy in the second group. Recurrence rates were lower in the second group (10.4%) compared to the first group (33.6%).

In the subsequent investigation conducted by Demircan et al. [10], a total of 60 patients aged 15-46 years were enrolled. The control group received the Karydakis flap procedure, while the intervention group underwent laser hair removal two weeks before and three weeks following the surgical operation. The rate of recurrence after one year was unexpectedly lower among the participants in the surgery group (4%) in comparison to those in the surgery and laser hair removal group (20%). This finding increased statistical heterogeneity and stood in stark contrast to all previous research conducted thus far.

Ghnnam and Hafez [11] performed a prospective randomized study with 86 patients. All patients received surgical excision with open wound healing by secondary intention. After the surgical site had healed, laser epilation was administered to one group, while the other group was subjected to mechanical or chemical hair removal techniques. Following the completion of the healing process at the surgical site, an average of four sessions for laser hair removal were carried out with only minimal discomfort being reported as an adverse effect. Follow-up was done till 2 +/- 1 years. The recurrence rate in the laser epilation group was 0 compared to the group that did not receive laser therapy.

Discussion

The recurrence rates in PSD continue to pose a formidable challenge that demands substantial resources to manage. Several methods have been used alone or in conjunction with surgical management options [3]. However, there is currently no established protocol to help decrease the frequency of recurrences in pilonidal disease, a condition that significantly affects the lives of patients. The proposed theories surrounding the shared concept of acquired hair insertion in the pathophysiology of PSD have led to the recommendation of removing hair either before or following surgical management [1,2]. Our study aimed to provide further insight into the subject matter and to assess the effectiveness of laser hair removal in minimizing the reoccurrence of pilonidal disease.

A limited number of RCTs have been carried out with a specific focus on the problem of recurrence. Following a thorough examination of the literature as outlined in the search methodology, we identified three RCTs that were closely pertinent to our research. The primary outcome was defined as the occurrence of PSD recurrence within a year. This encompassed the development of a new pilonidal abscess, folliculitis, or draining sinus after treatment, which necessitated antibiotic therapy, additional surgical incision and drainage, or excision. It was not possible to quantitatively analyze secondary outcomes due to the paucity of data surrounding these. Based on the available data, the group that received laser hair removal experienced significantly reduced recurrence rates in comparison to the group that exclusively relied on mechanical or chemical hair removal techniques [4,10,11].

Following block randomization, Minneci et al. [9] assigned patients to a control or an intervention group. The control group received standard education and training about hair removal in addition to a demonstration of mechanical hair removal in an intergluteal cleft. Patients and families in the control group were also provided with enough supplies to perform hair removal for six months, with an option to schedule a further appointment within this period. In addition to this, patients in the intervention group have their Fitzpatrick skin type assessed during their first assessment. They subsequently undergo laser depilation treatment every four to six weeks for a total of five sessions. Patients with Fitzpatrick skin types I-IV received an 810 nm application, while those with Fitzpatrick types V-VI received an Nd:YAG 28-joule application with an auto-pulse duration of 400 ms. There were no notable variances observed among intervention and control groups in terms of secondary outcomes, such as patient disability days, caregiver disability days, HRQOL scores, healthcare satisfaction scores, or perceived stigma.

The study published by Demicran et al. [10] used simple randomization to assign 30 patients to a control group and another 30 patients to the intervention group. The control group received a Karydakis flap reconstruction, while the intervention group underwent two sessions of laser hair epilation in addition to this. The sessions took place two weeks before and three weeks following the surgical intervention. An Alexandrite laser with a wavelength of 755 nm was used at 14-20 J/cm^2^ fluence, 18 mm spot size, and 3 ms pulse duration. There was no reported statistical difference in the two groups in terms of wound site infection symptoms, wound separation, and abscess formation at one week, one month, and three months.

Ghnnam and Hafez [11] subjected all enrolled patients aged 16-42 years to surgical excision of their pilonidal disease, which were left to heal by secondary intention. However, the intervention group also received four Alexandrite laser hair removal sessions at monthly intervals following the healing of their wound in addition to a Fusicort cream regimen applied to the treated skin twice a day for five days. The control group underwent traditional hair removal methods consisting of razor blade shaving and cream depilation. The researchers also assessed the adherence of patients to conventional hair removal methods, revealing that the majority of patients discontinued the use of mechanical or chemical hair removal techniques within the first year. 

A promising new minimally invasive approach that aims to reduce recurrence involves the use of a fistuloscope to enter and debride the sinus tract followed by thorough ablation. When compared to open excisional methods, the removal of hair, debris, and granulation tissue from all sinus tracts through endoscopic pilonidal sinus treatment (EPSiT) results in reduced recurrence rates and faster healing by minimizing the damage inflicted to surrounding healthy tissue [12,13].

Other novel treatment modalities for PSD

In addition to invasive and minimally invasive treatment modalities, lifestyle modifications play an essential role in preventing recurrence and should always be employed as a complementary measure to laser hair epilation. Adequate personal hygiene, such as consistent cleansing and preventing excessive moisture in the intergluteal cleft plays a crucial role in the prevention of infection and minimizing the likelihood of recurrence. PSD is widely recognized to be associated with obesity. By maintaining an optimal body mass index through a well-balanced diet and consistent physical activity, the depth of the natal cleft can be reduced. Consequently, this diminishes the likelihood of hair becoming embedded in the skin and subsequently leading to recurrence. Furthermore, extended periods of sitting can lead to heightened pressure and friction in the natal cleft region. Patients should be advised to incorporate regular breaks, utilize cushions for pressure relief, and refrain from prolonged sitting [14].

Another treatment adjunct that could assist in the reduction of pilonidal sinus recurrence is negative pressure wound therapy, also known as vacuum-assisted closure (VAC). As its name suggests, NPWT creates a vacuum at the wound site, applied via an occlusive dressing connected by tubing to a vacuum pump. The resulting negative pressure inside the wound bed speeds wound closure by reducing edema, improves perfusion through hydrostatic effects, and removes edema fluid and infectious material from the wound bed. Moreover, by promoting granulation tissue formation, NPWT facilitates healing which is essential for proper wound closure. It is now well established that NPWT can reduce the size of the wound and improve the rate of tissue repair, eliminating one of the main challenges associated with PSD: ensuring that the wound remains closed for the long term [15,16]. NPWT also reduces the number of required dressing changes, making postoperative outpatient management less painful and easier to adhere to [17].

The use of platelet-rich plasma (PRP) following surgical excision of pilonidal disease is an emerging innovative therapy that could further reduce recurrence rates by enhancing wound healing. The growth factors present in PRP play a crucial role in facilitating the process of epithelialization and collagen synthesis, which are vital for ensuring the long-lasting closure of wounds. The procedure entails centrifuging the patient's blood to separate the plasma, which is then applied to the sacral cleft. Growth factors present in PRP, such as platelet-derived growth factor (PDGF), transforming growth factor-beta (TGF-β), and vascular endothelial growth factor (VEGF), are vital in promoting angiogenesis, tissue repair, and anti-inflammatory processes [18]. Ahmed et al [19] conducted an additional study that illustrated how the integration of PRP with traditional surgical techniques led to improved outcomes. Patients who received PRP experienced shorter hospital stays, quicker return to normal activities, and a lower incidence of complications such as infection and wound dehiscence. 

This systematic review and meta-analysis have yielded encouraging findings supporting the use of laser hair epilation in the treatment of PSD. Nevertheless, it is imperative to emphasize certain limitations of the study. All included studies were at risk of bias, largely because of the inherent impracticality of blinding both participants and treating clinicians. Additionally, secondary outcomes could not be compared across the included studies. Furthermore, the authors only considered RCTs conducted in the English language.

## Conclusions

The authors offer a decisive recommendation in favor of laser hair epilation in PSD based on the systematic review and meta-analysis of all randomized controlled data to date on its use in the prevention of pilonidal sinus disease recurrence.

The authors suggest conducting additional high-quality trials investigating the role of laser hair epilation in PSD. Specifically, further trials that account for variation in skin and hair types should include a direct assessment of the ideal perioperative timing of individual laser hair epilation treatments in addition to evaluating the number of laser hair epilation sessions required to achieve the lowest recurrence rates possible.
